# Supplementary material for: A Critical View of the Application of the APEX Software (Aqueous Photochemistry of Environmentally-Occurring Xenobiotics) to Predict Photoreaction Kinetics in Surface Freshwaters
Source: Molecules. 2019 Dec 18;25(1):9. doi: 10.3390/molecules25010009 (PMC7017383; doi:10.3390/molecules25010009)
Supplement: Supplementary file 1 [file molecules-25-00009-s001.zip › 3.2.4_gcc-4.4.0.zip!/3.2.4_gcc-4.4.0/3.2.4_gcc-4.4.0/doc/octave/html/interpreter/Assignment-Ops.html]

Assignment Ops - Untitled


Next: Increment Ops,
Previous: Boolean Expressions,
Up: Expressions


---

### 8.6 Assignment Expressions

An assignment is an expression that stores a new value into a
variable. For example, the following expression assigns the value 1 to
the variable `z`:

```
     z = 1
```

After this expression is executed, the variable `z` has the value 1.
Whatever old value `z` had before the assignment is forgotten.
The = sign is called an assignment operator.

Assignments can store string values also. For example, the following
expression would store the value `"this food is good"` in the
variable `message`:

```
     thing = "food"
     predicate = "good"
     message = [ "this " , thing , " is " , predicate ]
```

(This also illustrates concatenation of strings.)

Most operators (addition, concatenation, and so on) have no effect
except to compute a value. If you ignore the value, you might as well
not use the operator. An assignment operator is different. It does
produce a value, but even if you ignore the value, the assignment still
makes itself felt through the alteration of the variable. We call this
a side effect.

The left-hand operand of an assignment need not be a variable
(see Variables). It can also be an element of a matrix
(see Index Expressions) or a list of return values
(see Calling Functions). These are all called lvalues, which
means they can appear on the left-hand side of an assignment operator.
The right-hand operand may be any expression. It produces the new value
which the assignment stores in the specified variable, matrix element,
or list of return values.

It is important to note that variables do *not* have permanent types.
The type of a variable is simply the type of whatever value it happens
to hold at the moment. In the following program fragment, the variable
`foo` has a numeric value at first, and a string value later on:

```
     octave:13> foo = 1
     foo = 1
     octave:13> foo = "bar"
     foo = bar
```

When the second assignment gives `foo` a string value, the fact that
it previously had a numeric value is forgotten.

Assignment of a scalar to an indexed matrix sets all of the elements
that are referenced by the indices to the scalar value. For example, if
`a` is a matrix with at least two columns,

```
     a(:, 2) = 5
```

sets all the elements in the second column of `a` to 5.

Assigning an empty matrix [] works in most cases to allow you to
delete rows or columns of matrices and vectors. See Empty Matrices.
For example, given a 4 by 5 matrix A, the assignment

```
     A (3, :) = []
```

deletes the third row of A, and the assignment

```
     A (:, 1:2:5) = []
```

deletes the first, third, and fifth columns.

An assignment is an expression, so it has a value. Thus, `z = 1`
as an expression has the value 1. One consequence of this is that you
can write multiple assignments together:

```
     x = y = z = 0
```

stores the value 0 in all three variables. It does this because the
value of `z = 0`, which is 0, is stored into `y`, and then
the value of `y = z = 0`, which is 0, is stored into `x`.

This is also true of assignments to lists of values, so the following is
a valid expression

```
     [a, b, c] = [u, s, v] = svd (a)
```

that is exactly equivalent to

```
     [u, s, v] = svd (a)
     a = u
     b = s
     c = v
```

In expressions like this, the number of values in each part of the
expression need not match. For example, the expression

```
     [a, b] = [u, s, v] = svd (a)
```

is equivalent to

```
     [u, s, v] = svd (a)
     a = u
     b = s
```

The number of values on the left side of the expression can, however,
not exceed the number of values on the right side. For example, the
following will produce an error.

```
     [a, b, c, d] = [u, s, v] = svd (a);
     -| error: element number 4 undefined in return list
```

A very common programming pattern is to increment an existing variable
with a given value, like this

```
     a = a + 2;
```

This can be written in a clearer and more condensed form using the
`+=` operator

```
     a += 2;
```

Similar operators also exist for subtraction (`-=`),
multiplication (`*=`), and division (`/=`). An expression
of the form

```
     expr1 op= expr2
```

is evaluated as

```
     expr1 = (expr1) op (expr2)
```

where op can be either `+`, `-`, `*`, or `/`.
So, the expression

```
     a *= b+1
```

is evaluated as

```
     a = a * (b+1)
```

and *not*

```
     a = a * b + 1
```

You can use an assignment anywhere an expression is called for. For
example, it is valid to write `x != (y = 1)` to set `y` to 1
and then test whether `x` equals 1. But this style tends to make
programs hard to read. Except in a one-shot program, you should rewrite
it to get rid of such nesting of assignments. This is never very hard.
